# Maternal methylmercury exposure through rice ingestion and child neurodevelopment in the first three years: a prospective cohort study in rural China

**DOI:** 10.1186/s12940-021-00732-z

**Published:** 2021-04-28

**Authors:** Sarah E. Rothenberg, Susan A. Korrick, Jihong Liu, Yanfen Nong, Hua Nong, Chuan Hong, Eva P. Trinh, Xu Jiang, Fred J. Biasini, Fengxiu Ouyang

**Affiliations:** 1 College of Public Health and Human Sciences, Oregon State University, Corvallis, OR USA; 2 Channing Division of Network Medicine, Department of Medicine, Brigham and Women’s Hospital and Harvard Medical School, Boston, MA USA; 3 Department of Environmental Health, Harvard T.H. Chan School of Public Health, Boston, MA USA; 4 Department of Epidemiology & Biostatistics, Arnold School of Public Health, University of South Carolina, Columbia, SC USA; 5 Maternal and Child Health Hospital, Daxin County, China; 6 Department of Environmental Health Sciences, Arnold School of Public Health, University of South Carolina, Columbia, SC USA; 7 Department of Psychology, University of Alabama at Birmingham, Birmingham, AL USA; 8 Present address: Centers for Disease Control and Prevention, Atlanta, GA USA; 9 Department of Psychology, University of South Carolina, Columbia, SC USA; 10 Present address: Department of Psychological Studies in Education, Temple University, Philadelphia, PA USA; 11 Ministry of Education and Shanghai Key Laboratory of Children’s Environmental Health, Xinhua Hospital, Shanghai Jiao Tong University School of Medicine, Shanghai, China

**Keywords:** Methylmercury, Neurodevelopment, Neurotoxicant, Rice consumption, Left-behind children

## Abstract

**Background:**

Rice is an important dietary source for methylmercury; however, rice does not contain the same beneficial nutrients as fish. Our main objective was to assess associations of prenatal methylmercury exposure through rice ingestion with child neurodevelopment in rural China.

**Methods:**

Eligible peripartum women were enrolled (*n* = 391), provided peripartum hair samples, and children’s neurodevelopment was assessed at 12 months (*n* = 264, 68%) and 36 months (*n* = 190, 48%) using the Bayley Scales of Infant Development, 2nd Edition, including the Mental Developmental Index (MDI) and the Psychomotor Developmental Index (PDI). Associations between prenatal methylmercury exposure during the third trimester [log_2_ maternal hair total mercury (THg)] and child’s neurodevelopment were assessed using linear mixed models for repeated measures.

**Results:**

In adjusted models, a doubling in maternal hair THg corresponded to a 1.3-point decrement in the MDI score [95% confidence interval (CI): − 2.6, − 0.14], and a 1.2-point decrement in the PDI score (95% CI: − 2.6, 0.14). Overall, adverse associations between maternal hair THg and MDI scores attenuated over time. However, associations were robust and stable over time among children whose primary caregiver was their parent(s). During the study follow-up, an increasing proportion of children were raised by grandparents (12 months: 9% versus 36 months: 27%), a trend associated with rural-to-urban parental migration for work.

**Conclusions:**

For young children living in rural China, a biomarker of prenatal methylmercury exposure was associated with decrements in cognitive function assessed between 12 and 36 months of age. Changes in the family structure over the study follow-up time interval potentially impacted children’s sensitivity to prenatal methylmercury exposure.

**Supplementary Information:**

The online version contains supplementary material available at 10.1186/s12940-021-00732-z.

## Introduction

For most populations, fish consumption is the main exposure pathway for methylmercury (MeHg), a potent neurotoxin [1]. Fish tissue is also a rich source of beneficial nutrients, including omega-3 fatty acids, which are critical for fetal brain development [2]. Disparate impacts on child neurodevelopment due to MeHg were reported by two of the longest running studies to date, among moderately exposed populations, in the Seychelles [3, 4] and the Faroe Islands [5]. Inconsistent findings were mainly attributed to differential confounding and/or effect modification by beneficial nutrients, which were more abundant in ocean fish, compared to lean whale meat, i.e., the main sources of MeHg ingested by the Seychelles and Faroese mothers, respectively [6, 7].

Rice is also a dietary source of MeHg, yet rice does not contain the same beneficial nutrients as fish [8]. In a comprehensive review of rice and mercury from 51 studies from 15 countries, MeHg comprised on average 30–36% of total mercury (THg) in rice grain [8]. Rice MeHg concentrations are lower compared to fish tissue; however, the high frequency of rice consumption in many populations for whom rice is a key dietary staple means that rice can be a major source of MeHg for many vulnerable populations [9–11].

There have been few studies, if any, concerning the impacts of prenatal MeHg exposure from maternal rice consumption on children’s neurodevelopment. In 2013, a prospective birth cohort study was initiated in Daxin County, Guangxi Province, China, to address this knowledge gap. Most study mothers ingested rice daily, but had no or infrequent fish consumption (Table 1) [10]. There were no nearby point sources of mercury pollution, such as coal-fired power plants or mercury mining, and rice MeHg concentrations in Daxin were considered low-level compared to more contaminated sites in China [8, 10]. Peripartum women were enrolled in Daxin County, and children’s neurodevelopment was assessed at 12 and 36 months. In previous analyses, we observed adverse associations between maternal MeHg exposure from pregnancy rice consumption and infant cognition at 12 months [14]. In the present study, we expanded on our previous work by assessing longer term MeHg impacts on child neurodevelopment through 36 months.

**Table 1:** Comparison of maternal/child characteristics between children completing the neurodevelopmental assessment and those who did not

		Follow-up at 12 months			Follow-up at 36 months		
	All (***n*** = 391) n (%)	No (***N*** = 127, 32%) n (%)	Yes (***N*** = 264, 68%) n (%)	***p***-value	No (***N*** = 201, 51%) n (%)	Yes (***N*** = 190, 49%) n (%)	***p***-value
**Mother’s Age upon Enrollment (years)**							
Age < 20	28 (7)	8 (6.3)	20 (7.6)	0.48	14 (7)	14 (7.4)	0.96
20 ≤ Age < 30	223 (57)	68 (54)	155 (59)		116 (58)	107 (56)	
30 ≤ Age < 45	140 (36)	51 (40)	89 (34)		71 (35)	69 (36)	
**Mother’s Ethnicity**							
Zhuang	333 (85)	104 (82)	229 (87)	0.28	168 (84)	165 (87)	0.58
Han	50 (13)	21 (17)	29 (11)		29 (14)	21 (11)	
Other	8 (2)	2 (1.6)	6 (2.3)		4 (2.0)	4 (2.1)	
**Mother’s Education Completed**							
< High School	316 (81)	110 (87)	206 (78)	0.04*	170 (85)	146 (77)	0.07
High School	47 (12)	9 (7.1)	38 (14)		19 (9.5)	28 (15)	
Some University	18 (4.6)	3 (2.4)	15 (5.7)		6 (3.0)	12 (6.3)	
Missing	10 (2.6)	5 (3.9)	5 (1.9)		6 (3.0)	4 (2.1)	
**Father’s Education Completed**							
< High School	304 (78)	103 (81)	201 (76)	0.22	164 (82)	140 (74)	0.02*
High School	57 (15)	14 (11)	43 (16)		25 (12)	32 (17)	
Some University	20 (5.1)	4 (3.1)	16 (6.7)		5 (2.5)	15 (7.9)	
Missing	10 (2.6)	6 (4.7)	4 (1.5)		7 (3.5)	3 (1.6)	
**Mother’s Occupation**							
Farmer	298 (76)	108 (85)	190 (72)	0.015*	158 (79)	140 (74)	0.50
Worker^**a**^	31 (7.9)	6 (4.7)	25 (9.5)		12 (6.0)	19 (10)	
Unemployed	42 (11)	9 (7.1)	33 (13)		21 (10)	21 (11)	
Other	13 (3.3)	1 (< 1)	12 (4.5)		7 (3.5)	6 (3.2)	
Missing	7 (1.8)	3 (2.4)	4 (1.5)		3 (1.5)	4 (2.1)	
**Father’s Occupation**							
Farmer	289 (74)	101 (80)	188 (71)	0.08	155 (77)	134 (71)	0.046*
Worker^**a**^	53 (14)	13 (10)	40 (15)		18 (9.0)	35 (18)	
Unemployed	23 (5.9)	5 (3.9)	18 (6.8)		14 (7.0)	9 (4.7)	
Other	17 (4.3)	2 (1.2)	15 (5.7)		8 (4.0)	9 (4.7)	
Missing	9 (2.3)	6 (4.7)	3 (1.1)		6 (3.0)	3 (1.6)	
**Household Monthly Income (RMB)**^**b**^							
Income < 2000	230 (59)	68 (54)	162 (61)	0.26	114 (57)	116 (61)	0.72
2000 ≤ Income < 5000	107 (27)	39 (31)	68 (26)		58 (29)	49 (26)	
Income ≥5000	19 (4.9)	8 (6.3)	11 (4.2)		10 (5.0)	9 (4.7)	
Missing	35 (9.0)	12 (9.4)	23 (8.7)		19 (9.5)	16 (8.4)	
**Maternal Pre-Pregnancy BMI (kg/m**^**2**^**)**^**c**^							
Underweight	90 (23)	21 (17)	69 (26)	0.07	39 (19)	51 (27)	0.33
Normal Weight	231 (59)	82 (65)	149 (56)		126 (63)	105 (55)	
Overweight	60 (15)	23 (18)	37 (14)		30 (15)	30 (16)	
Obese	9 (2.3)	1 (< 1)	8 (3.3)		5 (2.5)	4 (2.1)	
Missing	1 (< 1)	0 (0)	1 (< 1)		1 (< 1)	0 (0)	
**Maternal Smoking During Pregnancy**							
No	377 (96)	117 (92)	260 (98)	0.003**	191 (95)	186 (98)	0.37
Yes	5 (1.3)	5 (3.9)	0 (0)		4 (2.0)	1 (< 1)	
Missing	9 (2.3)	5 (3.9)	4 (1.6)		6 (3.0)	3 (1.6)	
**2nd-Hand Smoke Exposure During Pregnancy**							
No	214 (55)	65 (51)	149 (56)	0.41	100 (50)	114 (60)	0.06
Yes	163 (42)	56 (44)	107 (41)		92 (46)	71 (37)	
Missing	14 (3.6)	6 (4.7)	8 (3.3)		9 (4.5)	5 (2.6)	
**Alcohol During Pregnancy**							
No	379 (97)	121 (95)	258 (98)	1.0	196 (98)	183 (96)	0.36
Yes	4 (1.0)	1 (< 1)	3 (1.1)		1 (< 1)	3 (1.6)	
Missing	8 (2.0)	5 (3.9)	3 (1.1)		4 (2.0)	4 (2.1)	
**Anemia During Pregnancy**							
No	377 (96)	123 (97)	254 (96)	0.56	193 (96)	184 (97)	1.0
Yes	13 (3.3)	3 (2.4)	10 (3.8)		7 (3.5)	6 (3.2)	
Missing	1 (< 1)	1 (< 1)	0 (0)		1 (< 1)	0 (0)	
**Primipara**							
No	185 (47)	66 (52)	119 (45)	0.20	97 (48)	88 (46)	0.64
Yes	190 (49)	56 (44)	134 (51)		95 (47)	95 (50)	
Missing	16 (4.1)	5 (3.9)	11 (4.2)		9 (4.5)	7 (3.7)	
**Maternal Rice Consumption**							
< Daily	47 (12)	12 (9.4)	35 (13)	0.26	23 (11)	24 (13)	0.72
≥ Daily	323 (83)	109 (86)	214 (81)		167 (83)	156 (82)	
Missing	21 (5.4)	6 (4.7)	15 (5.7)		11 (5.5)	10 (5.3)	
**Maternal Fish Consumption (servings/week)**							
0 servings/week	169 (43)	60 (47)	109 (41)	0.33	87 (43)	82 (43)	0.87
0 < servings/week < 2	178 (46)	51 (40)	127 (48)		93 (46)	85 (45)	
≥ 2 servings/week	44 (11)	16 (13)	28 (11)		21 (10)	23 (12)	
**Cesarean Birth**							
No	285 (73)	91 (72)	194 (27)	0.70	138 (69)	147 (77)	0.05*
Yes	106 (27)	36 (28)	70 (73)		63 (31)	43 (23)	
**Child Sex**							
Male	198 (51)	53 (42)	140 (53)	0.04*	94 (47)	104 (55)	0.12
Female	193 (49)	74 (58)	124 (47)		107 (53)	86 (45)	
**Gestational Age (weeks)**							
37 ≤ Gestational Age < 39	151 (39)	55 (43)	96 (36)	0.22	81 (40)	70 (37)	0.54
39 ≤ Gestational Age < 41	215 (55)	68 (54)	147 (56)		110 (55)	105 (55)	
Gestational Age ≥ 41	22 (5.6)	4 (3.2)	18 (6.8)		9 (4.5)	13 (6.8)	
Missing	3 (< 1)	0 (0)	3 (1.1)		1 (< 1)	2 (1.1)	
**Birth weight-for-gestational age z-score (centile)**^**d**^							
Value < 10th	45 (12)	13 (10)	32 (12)	0.41	20 (10)	25 (13)	0.47
10th ≤ Value <90th	327 (84)	111 (87)	216 (81)		173 (86)	154 (81)	
Value ≥90th	16 (4.1)	3 (2.4)	13 (4.9)		7 (3.5)	9 (4.7)	
Missing	3 (< 1)	0 (0)	3 (1.1)		1 (< 1)	2 (1.1)	
**Questionnaire Responses at 12 or 36 Months**							
**Primary Caregiver**							
Mother	NA	NA	208 (79)	NA	NA	115 (61)	NA
Father	NA	NA	32 (12)		NA	23 (12)	
Grandparent	NA	NA	24 (9.1)		NA	52 (27)	
Other relative	NA	NA	0 (0)		NA	0 (0)	
**Mother or Father is a Migrant Worker**							
No	NA	NA	251 (95)	NA	NA	149 (78)	NA
Yes	NA	NA	9 (3.5)		NA	41 (22)	
Missing	NA	NA	4 (1.5)			0 (0)	
**Older Child in the Household**							
No	NA	NA	160 (61)	NA	NA	102 (54)	NA
Yes	NA	NA	104 (39)		NA	86 (45)	
Missing	NA	NA	0 (0)		NA	2 (1.1)	
**Breastfeeding Duration > Median (8.5 months)**^**e**^							
No	NA	NA	127 (48)	NA	NA	89 (47)	NA
Yes	NA	NA	136 (52)		NA	100 (53)	
Missing	NA	NA	1 (< 1)		NA	1 (< 1)	

*p ≤ 0.05, **p < 0.01, *p*-values are for chi-squared test or Fisher’s exact test between non-missing categories

*BMI* body mass intake, *RMB* ren min bi = Chinese currency

^a^Workers include: civil servant, white-collar worker, skilled worker, unskilled worker, and shopkeeper

^b^Between 2013–2014, 2000 RMB = US$324, US$5000 RMB = $810

^c^BMI for Asian populations: underweight (BMI < 18.5 kg/m2), normal weight (18.5 kg/m2 ≤ BMI < 23 kg/m2), overweight (23 kg/m2 ≤ BMI < 27.5 kg/m2), and obese (BMI ≥ 27.5 kg/m2) [12]

^d^Birth weight-for-gestational age z-scores were calculated using the Intergrowth-21 Newborn Birth standards, which were based on a reference population of 20,486 births from eight countries including 17% from China [13]

^e^Median breastfeeding duration based on 12-month and 36-month responses (*n* = 332 mothers)

## Methods

### Enrollment and follow-up

The study is located in Daxin County, which is predominantly rural, with a population of 359,800 [10, 14]. Approximately 50,000 county residents live in the town of Daxin, and most of the remaining are rice farmers who reside outside the town.

Cohort enrollment occurred between May 2013 and March 2014, during which time 1261 women gave birth at the Maternal and Child Health Hospital in Daxin County. Mothers were enrolled up to 4 weeks before delivery or within 1 week postpartum; study eligibility requirements included being in good general health, residing in Daxin County during the previous 3 months, and planning to remain for the next 12 months. Of the 1261 births, 574 (46%) were ineligible because mothers resided outside Daxin County, and an additional 228 mothers (18%) were ineligible due to infectious disease (e.g., Hepatitis B). Of the remaining 459 eligible mothers, 51 (11%) refused participation, and 408 (89%) enrolled in the study. Ten mothers were subsequently excluded because they actually lived outside Daxin County (*n* = 3), gave birth to twins (*n* = 1), or data collection was incomplete (*n* = 6). For this analysis, seven mothers with pre-term infants (< 37 weeks gestation) were also excluded, resulting in a final analysis cohort of 391 mother-infant pairs (See Supplementary Figure 1, Additional file 1).

### Collection of exposure biomarkers and other biosamples

During the peripartum period, while in the hospital, a maternal hair sample was collected from the occipital region (~ 50 strands). Hair THg, a proxy biomarker of MeHg exposure [15], was analyzed in the proximal hair section corresponding to exposures during the third trimester of pregnancy, as determined for Asian women (3.4 cm) [10]. Two non-fasting maternal blood samples were collected, including one for analysis of lead (Pb) (in whole blood collected with lithium heparin anticoagulant), and one for analysis of serum selenium (Se), zinc (Zn), and fatty acids (with serum separated by centrifugation). A family member brought a rice sample from home for analysis of MeHg. THg concentrations were analyzed in 13 commonly consumed freshwater fish purchased in the town of Daxin [10]. A comprehensive literature review was used to determine the fish tissue THg concentrations for other varieties of fish queried on the study food frequency questionnaire (FFQ) [10].

### Lab analyses

Detailed lab analyses and quality assurance/quality control are provided in Supplementary Materials and Supplementary Table 1, in Additional file 1. Rice MeHg concentrations were analyzed according to Liang et al. [16] and U.S. Environmental Protection Agency (U.S. EPA) Method 1630, using cold vapor-atomic absorption spectrometry [17]. THg concentrations in maternal hair and fish tissue were analyzed following U.S. EPA Method 7473, using atomic absorption spectrometry [18]. Blood Pb levels were analyzed directly using graphite furnace-atomic absorption spectrometry [19]. Serum Zn and Se concentrations were analyzed by inductively coupled plasma-mass spectrometry, following U.S. EPA 3050B [20]. Average %recoveries of standard reference materials and matrix spikes ranged from 85 to 115%. The relative standard deviation between sample replicates ranged from 4.2–7.7% for THg and MeHg analyses, and < 20% for other metals in whole blood and serum. All measurements exceeded the limits of detection.

Maternal serum fatty acids [omega-3: docosahexaenoic acid (DHA) and eicosapentaenoic acid; omega-6: alpha-linolenic acid, linoleic acid and arachidonic acid (AA)] were assessed by gas-liquid chromatography.

Hair THg and rice MeHg were analyzed at the Rothenberg Mercury Lab (University of South Carolina, Columbia, South Carolina, USA), fish tissue THg was analyzed at the Beijing Lumex Analytical Co. Ltd., other metals in blood and serum were analyzed at the State Key Lab for Children’s Environmental Health in Shanghai, China, and serum fatty acids were analyzed at the State Key Laboratory of Nutrition and Metabolism in Shanghai, China.

### Questionnaire data collection

While in the hospital, mothers completed a detailed questionnaire concerning demographics, education, income, pregnancy history (including cigarette and alcohol use), and their infant’s birth weight and sex. Mothers also completed a modified semi-quantitative 102-item FFQ, reflecting their dietary intake during the third trimester, which was previously validated among pregnant mothers in rural China [21]. Methods for ascertaining servings per day, serving size, and calculation of energy intake (kcal) are in Supplementary Materials in Additional file 1. Daily MeHg intake from rice (or fish) (μg/day) was calculated by multiplying the rice MeHg concentration (or fish tissue THg concentration) (μg/g) × ingestion rate (g/day); MeHg intake from rice and fish were summed, and the proportional intake of MeHg from each dietary source was determined [10].

At the 12- and 36-month follow-up visits, parents or caregivers completed an interviewer administered questionnaire regarding the child’s health, breastfeeding duration, primary caregiver (mother, father, grandmother, grandfather, or other relative), and diet in the previous 24 h (36 months only). We also asked whether parents worked outside Daxin County. Economic growth in China has created large-scale rural-to-urban migration to alleviate family poverty. Anyone living in a location outside their permanent residence (called *hukou* in Chinese) is considered a migrant worker. In 2017, China’s migrant worker population was 244 million, accounting for 17.6% of the total population [22]. At 12 months, the parent/primary caregiver was asked “How many months did the child live with both parents?” We assumed if the answer was less than the child’s age minus 3 months (i.e., < 9 months for a child 12.0 months of age), at least one parent worked outside Daxin County. At 36 months, more direct questions were asked of each parent: “In the last 12 months, did the child’s father (or mother) work outside Daxin County (for at least three months)? If yes, how many months?”

Children’s length/height and weight were measured by hospital staff at 12 months using a digital infant scale/length board (Model # WS-RTG-1GD, Shanghai, China) and at 36 months using a digital standing scale/stadiometer (Model # HCS-100-RT, Jiangsu, China). Z-scores for weight-for-age, length/height-for-age, and weight-for-length/height were calculated using the 2006 World Health Organization child growth standards [23], with the R package “anthro” (21 May 2020).

### Neurodevelopmental assessment

At 12 and 36 months, study children were assessed with the Bayley Scales of Infant Development, 2nd Edition (BSID-II), which was translated into Chinese. Of the 391 infants enrolled at birth and considered for this analysis, 311 returned for the 12-month BSID-II and 190 returned for the 36-month BSID-II. Neurodevelopmental data were not analyzed for those who were outside a nine-week window of the targeted exam age (*n* = 47 at 12 months and *n* = 0 at 36 months) resulting in 264 (68%) children with 12-month assessments and 190 (48%) children with 36-month assessments (See Supplementary Figure 1, Additional file 1).

The BSID-II yields two summary performance measures: the Mental Developmental Index (MDI) and the Psychomotor Developmental Index (PDI), which are age-standardized to a mean of 100 and standard deviation (SD) of 15, based on an English-speaking U.S. reference population [24]. The BSID-II underwent pilot testing on children from Daxin County prior to administration to study participants, and the same examiner, who spoke the local dialect, administered the BSID-II to all children at both time points (co-author YN). The examiner was trained in Bayley administration by co-authors XJ (12 months) and EPT (36 months), who are developmental psychologists. Examiner reliability was assessed throughout the follow-up period by videotaping a subset of exams (*n* = 8 children at 12 months, *n* = 5 children at 36 months). Both MDI and PDI sections were viewed and re-scored by co-authors EPT and FJB (also a developmental psychologist), and differences in scoring were minor.

### Data analysis

Our goal was to determine the associations between neurodevelopment (assessed with the BSID-II) and a biomarker of prenatal MeHg exposure (maternal hair THg). Potential nonlinear associations were evaluated using generalized additive models (R package “gam”; July 20, 2018) with smoothing splines computed using four degrees of freedom [25]. Two sets of models were developed, one for each time point (12 and 36 months) (See Supplementary Figure 2, Additional file 1). An approximate F-test was used to determine whether including the nonlinear component of the smooth term resulted in a better model fit than assuming a linear relationship. There were no strong non-linear relationships (F-test, *p* = 0.08–0.70); however, the *p*-value for the smooth term in the PDI GAM model at 12 months was 0.08. When one outlying observation was removed (maternal hair THg = 1.7, PDI = 105), the smooth term *p*-value increased to 0.30.

As there was no strong evidence of non-linear relationships, we used a linear mixed model to analyze repeated test scores (at 12 and 36 months) on each child. Maternal hair THg was log_2_-transformed to improve normality of the residuals. The model was adjusted for within-person correlation, and included an indicator variable for time (12 months versus 36 months). We also investigated the potential for MeHg associations to vary over time, by including the interaction between MeHg and time in our models. Because we observed differences over time in the association between MeHg and MDI scores, we investigated the potential for MeHg associations to vary by other critical time-varying factors. In particular, we assessed the potential for associations to vary by caregiver (in separate models for each exam time), by including the interaction between MeHg and caregiver (2 categories: parent or grandparent).

Unless otherwise noted, all models were adjusted for the same covariates as those in our prior analysis of the 12-month BSID-II [14], which was based on the relationship between each covariate and outcome measures; added variable plots; evidence of confounding of effect estimates for MeHg; and comparison of Akaike Information Criterion between models with/without covariates [26]. These covariates included maternal age (years), maternal fish consumption (0 servings/weekly, 0 < servings/weekly< 2 servings/weekly, or ≥ 2 servings/weekly), maternal rice consumption (<daily, or ≥ daily), maternal serum Zn, log_2_ maternal blood Pb, log_2_ maternal energy intake (kcal), pre-pregnancy body mass index (BMI) (underweight, normal weight, or overweight/obese), maternal education completed (<high school, high school, or some university), child sex, child’s age at testing (months), and birth weight-for-gestational age (z-score) [14]. We considered all of the other factors in Tables 1 and 2; however, they did not contribute to model fit and did not change the effect estimate for MeHg exposure. Missing data on covariates were imputed using multiple imputation based on the multivariate normal distribution [27], conditional on parental and child characteristics, and maternal biomarker concentrations (Tables 1 and 2) [14]. Because of co-linearity with time in the linear mixed models, child’s age at testing was replaced with the difference between child’s age at testing and the targeted age (months). Additionally, a time varying indicator of the child’s primary caregiver (three categories: mother, father, or grandparent) was included. We also considered an indicator of whether at least one parent was a migrant worker; however, due to multicollinearity with the child’s primary caregiver (See Supplementary Table 2, Additional file 1), only the primary caregiver remained in the model (it had the higher partial r-squared). Regression diagnostics for the linear mixed models included examination of residual plots, and assessment of potential influential observations. Assumptions for model residuals were checked (no evidence of non-linearity, constant variance, normal distribution).

**Table 2:** Comparison of maternal biomarkers and maternal diet between returning and non-returning participants (*n* = 391 mother/child pairs)

		Follow-up at 12 months			Follow-up at 36 months		
	All Median (Range)	No Median (Range)	Yes Median (Range)	***p***-value	No Median (Range)	Yes Median (Range)	***p***-value
**N**	**391**	**127**	**264**		**201**	**190**	
**Hair THg (**μg**/g)**	0.40 (0.08, 1.7)	0.41 (0.08, 1.3)	0.39 (0.08, 1.7)	0.53	0.38 (0.08, 1.3)	0.42 (0.12, 1.7)	0.12
**Rice MeHg (ng/g)**	2.2 (0.32, 15)	2.3 (0.53, 9.7)	2.1 (0.32, 15)	0.37	2.3 (0.34, 13)	2.1 (0.32, 15)	0.45
**N**	**389**	**127**	**262**		**200**	**189**	
**%MeHg intake from rice**	87 (0, 100)	91 (0, 100)	82 (0.30, 100)	0.15	87 (0.30, 100)	86 (0, 100)	0.33
**N**	**390**	**126**	**264**		**200**	**190**	
**Serum Zn (μg/L)**	715 (344, 1009)	714 (543, 1009)	715 (344, 905)	0.25	711 (344, 1009)	720 (575, 960)	0.20
**Blood Pb (μg/dL)**	2.6 (0.96, 8.6)	2.7 (1.2, 8.6)	2.6 (0.96, 7.8)	0.17	2.6 (0.96, 8.6)	2.7 (1.1, 7.8)	0.55
**Serum DHA (mg/mL)**	0.09 (0.04, 0.33)	0.09 (0.04, 0.18)	0.09 (0.04, 0.33)	0.25	0.09 (0.04, 0.19)	0.09 (0.04, 0.33)	0.20
**Serum EPA (mg/mL)**	0.007 (0.001, 0.09)	0.007 (0.001, 0.08)	0.007 (0.001, 0.09)	0.63	0.007 (0.001, 0.09)	0.008 (0.002, 0.07)	0.24
**Serum N-6/N-3 (unitless)**	12 (3.5, 25)	12 (5.3, 24)	11 (3.5, 25)	0.27	12 (3.5, 24)	12 (5.0, 25)	0.49
**N**	**389**	**126**	**263**		**199**	**190**	
**Serum Se (μg/L)**	152 (66, 535)	148 (74, 274)	153 (66, 535)	0.25	150 (66, 535)	153 (69, 323)	0.49
**N**	**376**	**121**	**255**		**194**	**182**	
**% Calories from fat**	33 (13, 81)	33 (16, 80)	33 (13, 81)	0.89	34 (18, 80)	32 (13, 81)	0.02*
**% Calories from carbohydrates**	56 (12, 78)	57 (14, 78)	55 (12, 75)	0.53	54 (12, 73)	56 (12, 78)	0.05*
**% Calories from protein**	11 (5.1, 25)	10 (5.2, 18)	12 (5.1, 25)	< 0.001***	11 (5.2, 25)	11 (5.1, 22)	0.26
**Total Energy Intake (kcal)**	1995 (515, 4637)	1863 (515, 4637)	2052 (549, 4438)	0.02*	2000 (515, 4535)	1989 (549, 4637)	0.38

*p ≤ 0.05, *** *p* < 0.001 *p*-values are for Wilcoxon rank sum test

*DHA* docosahexaenoic acid, *EPA* eicosapentaenoic acid, *MeHg* methylmercury, *N-6 fatty acids* linoleic acid and arachidonic acid, *N-3 fatty acids* DHA, EPA and alpha-linolenic acid, *Pb* lead, *Se* selenium, *THg* total mercury, *Zn* zinc

As sensitivity analyses, we investigated models: (1) limiting to children examined at both time points to determine whether differences in MeHg associations with Bayley scores between 12 and 36 months were due to differential loss to follow up; (2) using the raw Bayley scores; (3) including only participants with complete data; (4) including only participants who were non-fish eaters; (5) stratifying models by sex; (6) stratifying by median breastfeeding duration; and (7) without adjustment for covariates.

Analyses were performed using Stata (Version 16.0, College Station, TX, USA), and the R-platform (Version 3.5.3, 11 March 2019).

## Results

A total of 305 children had at least one BSID-II assessment at 12 months and/or 36 months. One-hundred and forty-nine (49%) of these 305 children were assessed at both 12 and 36 months. Some differences were noted between those participating in Bayley testing and those who did not (Tables 1 and 2). Compared to those who did not participate, children evaluated at 12 or 36 months had at least one parent who was more educated, and was less likely to be a rice farmer and more likely to be a worker (e.g., shop keeper). At 12 months, mothers of those evaluated were less likely to smoke during pregnancy compared to non-participants; in addition, a higher proportion of male children were assessed. At 36-months, mothers were less likely to have a cesarean birth, compared to non-returning participants. Lastly, compared to those lost to follow-up, at 12 months, mothers consumed proportionately more protein and their total energy intake (kcal) was higher, while at 36 months, mothers consumed proportionately less fat and more carbohydrates (Table 2).

The median maternal hair THg concentration among the Daxin mothers was 0.40 μg/g (range: 0.08, 1.7 μg/g; Table 2). Study participants’ MeHg exposure was primarily from rice, with a median proportional intake from rice of 87% (Table 2) [10].

The percentage of children who did not live with both parents for at least 3 months within the year prior to the exam increased between 12 and 36 months from 3.5% (*n* = 9/260) to 22% (*n* = 41/190) (Table 1). At both 12 and 36 months, children with at least one migrant worker parent were more likely to have a grandparent as the primary caregiver, while mothers were more likely to be the primary caregiver in families without a migrant parent (See Supplementary Table 2, Additional file 1). As a result, 9% (24/264) of study 12-month olds, and 27% (52/190) of study 36-month-olds had at least one grandparent as the primary caregiver (Table 1). Family structure correlated with other predictors of child development (See Supplementary Tables 2 and 3, Additional file 1). For example, at 12 months, children with a grandparent (versus a parent) as the primary caregiver were breastfed for shorter periods, and were born to mothers who were less likely to be underweight, and whose pregnancy diet had proportionately fewer calories from protein.

At 12 and 36 months, the mean (± SD) standardized MDI scores were 99 ± 9.8 (range: 66–120) and 86 ± 9.2 (range: 62–106), respectively, while the mean (± SD) standardized PDI scores were 88 ± 11 (60–121) and 93 ± 12 (range: 71–122), respectively (Table 3). In longitudinal studies, a decline in BSID-II MDI and/or PDI scores over time has been observed among other non-English speaking cohorts in Nepal [28], Japan [29], and the Seychelles [30]. Pearson’s correlation between the MDI and PDI scores was 0.37 at 12 months, and 0.62 at 36 months, which were comparable to the correlation coefficients reported for test standardization data among U.S. children (12-month r = 0.38, 36-month r = 0.56) [24]. Although average MDI scores declined between 12 and 36 months, MDI scores were moderately correlated over time (r = 0.30), whereas PDI scores were not (r = 0.10).

**Table 3:** Standardized scores for Bayley Scales of Infant Development, 2nd Edition among study participants

		n	Median	Mean	SD	Range
**MDI**	**12-month**	264	99	99	9.8	66–120
	**36-month**	190	86	86	9.2	62–106
**PDI**	**12-month**	264	86	88	11	60–121
	**36-month**	190	92	93	12	71–122

*MDI* Mental Developmental Index, *PDI* Psychomotor Developmental Index

Compared to mothers who never or rarely ingested fish, MDI scores increased by 4.7 points [95% confidence interval (CI): 1.4, 8.0], while PDI scores increased by 4.0 points (95% CI: 0.23, 7.7), among children whose mothers ingested ≥2 servings of fish/weekly (Table 4). The average or median fish/shellfish THg concentrations were considered low-level (10–74 ng/g) [10], supporting the notion that maternal ingestion of low-mercury fish during pregnancy benefits children’s neurodevelopment [3, 31, 32]. Compared to children cared for by their mothers, MDI scores were lower among children cared for by a grandparent, and MDI scores were lower among boys compared to girls. Both MDI and PDI scores were higher among children born to normal weight mothers, compared to underweight mothers. Although all children were assessed within a 9-week window for the targeted test date and all scores were age-standardized, older children had better MDI and PDI scores.

**Table 4:** Linear mixed models investigating associations between children’s neurodevelopment and prenatal methylmercury exposure, including the adjusted regression coefficients (95% confidence interval) (*n* = 454 observations)

	Base model				Fully adjusted model			
	MDI (standardized scores)		PDI (standardized scores)		MDI (standardized scores)		PDI (standardized scores)	
	Beta	95% CI	Beta	95% CI	Beta	95% CI	Beta	95% CI
**Log**_**2**_ **Maternal** **Hair THg (μg/g)**	−1.4	(−2.8, 0.11)	−1.1	(−2.7, 0.62)	−1.7	(−3.1, −0.26)*	− 1.2	(− 2.9, 0.43)
**Log**_**2**_ **Maternal** **Hair THg (**μg**/g) × time (12 or 36 months)**	0.89	(− 1.2, 3.0)	0.16	(− 2.5, 2.9)	0.91	(− 1.2, 3.0)	0.01	(− 2.7, 2.7)
**Exam time**								
12 months	Referent		Referent		Referent		Referent	
36 months	−12	(− 15, −8.8)***	5.0	(1.0, 8.9)*	− 12	(− 15, −8.5)***	5.1	(1.1, 9.1)**
**Maternal Age (yr)**					−0.009	(− 0.17, 0.15)	− 0.08	(− 0.26, 0.11)
F**ish consumption (servings/week)**	NA		NA					
0 servings/week					Referent		Referent	
0 < servings/week < 2					1.7	(−0.34, 3.7)	0.89	(−1.4, 3.2)
≥ 2 servings/week					4.7	(1.4, 8.0)**	4.0	(0.23, 7.7)*
**Maternal Rice Consumption**	NA		NA					
< Daily					Referent		Referent	
≥ Daily					−2.7	(−5.9, 0.58)	0.24	(−3.5, 3.9)
**Maternal serum Zn**	NA		NA		0.01	(−0.003, 0.03)	0.007	(−0.009, 0.02)
**Log**_**2**_ **Maternal** **Blood Pb (μg/dL)**	NA		NA		0.11	(−2.0, 2.2)	−0.95	(−3.3, 1.4)
**Log**_**2**_ **Maternal** **Energy Intake (kcal)**	NA		NA		1.7	(−0.31, 3.8)	2.3	(−0.07, 4.6)
**Maternal pre-pregnancy BMI**	NA		NA					
Underweight					Referent		Referent	
Normal Weight					3.2	(0.99, 5.4)**	2.6	(0.13, 5.1)*
Overweight or Obese					2.1	(−0.73, 5.0)	2.6	(−0.64, 5.8)
**Maternal Education Completed**	NA		NA					
< High School					Referent		Referent	
High School					0.24	(−2.4, 2.9)	−0.89	(−3.9, 2.1)
Some University					3.2	(−0.81, 7.1)	−0.39	(−4.9, 4.1)
**Child Sex (1 = male)**	NA		NA		−1.9	(−3.7, −0.04)*	−1.9	(−4.0, 0.13)
**Primary Caregiver**	NA		NA					
Mother					Referent		Referent	
Father					0.95	(−1.6, 3.5)	−0.89	(−4.0, 2.2)
Grandparent					−2.9	(−5.2, −0.45)*	−2.3	(−5.2, 0.60)
**Birth weight-for-gestational age** **(z-score)**	NA		NA		−0.18	(−1.3, 0.92)	0.64	(−0.60, 1.9)
**Child’s age difference (months)**	NA		NA		2.1	(0.69, 3.5)**	2.0	(0.30, 3.7)*

*p < 0.05, **p < 0.01, ***p < 0.001, *p*-value is for the Beta coefficient

*BMI* body mass intake, *CI* confidence interval, *MDI* Mental Developmental Index, *NA* not applicable, *Pb* lead, *PDI* Psychomotor Developmental Index, *THg* total mercury, *Zn* zinc

In adjusted linear mixed models, a doubling in maternal hair THg corresponded to a 1.3-point decrease in the MDI score (95% CI: − 2.6, − 0.14) (Table 5). Similarly, a doubling in maternal hair THg corresponded to a 1.2-point decrease in the PDI score (95% CI: − 2.6, 0.14), although the CI included the null.

**Table 5:** Adjusted regression coefficients (95% confidence interval), from linear mixed models, including exam time specific associations

		Time Specific Associations		
	All observations^**a**^ (***n*** = 454)	12 months (***n*** = 264)	36 months (***n*** = 190)	Interaction term ***p***-value
**MDI** **Log**_**2**_ **Hair THg (β)**	−1.3 (−2.6, −0.14)*	− 1.7 (−3.1, − 0.26)*	−0.77 (− 2.5, 1.0)	0.39
**PDI** **Log**_**2**_ **Hair THg (β)**	−1.2 (− 2.6, 0.14)	−1.2 (− 2.9, 0.43)	−1.2 (− 3.5, 1.0)	0.99

*p < 0.05, *p*-values are for the Beta coefficients

*MDI* Mental Developmental Index, *PDI* Psychomotor Developmental Index, *THg* total mercury

Note: All estimates are from models that were adjusted for maternal age (years), maternal fish consumption (0 servings/weekly, 0 < servings/weekly< 2 servings/weekly, or ≥ 2 servings/weekly), maternal rice consumption (<daily, or ≥ daily), maternal serum zinc (μg/L), log_2_ maternal blood lead (μg/dL), log_2_ maternal energy intake (kcal), pre-pregnancy body mass index (underweight, normal weight, or overweight/obese), maternal education completed (<high school, high school, or some university), child sex, birth weight-for-gestational age (z-score), child’s primary caregiver (3 categories: mother, father, or grandparent), the difference in child’s age between the targeted age and actual age at testing (months), a categorical variable for exam time (12 or 36 months), and the interaction between THg and exam time

^a^This model does not include the interaction of THg with exam time

Although adverse associations between MeHg and Bayley scores were observed at both exams, associations with MDI were attenuated at 36 months compared to 12 months (Table 5). We assessed whether changes in family structure over time may have contributed to the apparent change in MeHg-Bayley associations. Among children primarily cared for by a parent, MeHg-associated decrements in MDI were similar at 12 and 36 months (even slightly stronger at 36 months compared to 12 months) with respective declines of − 1.3 (95% CI: − 2.9, 0.20) and − 1.6 (95% CI: − 3.7, 0.54) per doubling of maternal hair THg (Table 6). In addition, among children primarily cared for by a parent, MeHg-associated decrements in PDI were similar at 12- and 36-months with respective declines of − 1.5 (95% CI: − 3.3, 0.29) and − 1.9 (− 4.7, 0.79), respectively (Table 6). However, with one exception, there was no evidence of an adverse association of MeHg with Bayley MDI or PDI performance at either age among children cared for by grandparents. The one exception was an enhanced adverse MeHg-Bayley MDI relationship at 12 months among the 24 children being cared for primarily by a grandparent(s) (β = − 5.7, 95% CI: − 9.8, − 1.6) (Table 6). This analysis also included unexpected evidence of a potential beneficial association of MeHg with 36-month MDI among children cared for by a grandparent(s) (β = 3.1, 95% CI: − 0.21, 6.3). This latter observation likely contributed to the attenuation of the overall MeHg-MDI association over time.

**Table 6:** Adjusted regression coefficients (95% confidence interval), including child’s primary caregiver-specific associations

12 months				
	**Mothers, Fathers, & Grandparents**^**a**^ **(*n*** **= 264, 100%)**	**Caregiver specific association**		
		**Mothers/Fathers** **(*n*** **= 240, 91%)**	**Grandparents** **(*n*** **= 24, 9%)**	**Interaction term** ***p*-value**
**MDI** **Log**_**2**_ **Hair THg (β)**	−1.9 (− 3.3, − 0.43)*	−1.3 (− 2.9, 0.20)	− 5.7 (−9.8, − 1.6)**	0.052
**PDI** **Log**_**2**_ **Hair THg (β)**	−1.2 (− 2.9, 0.49)	−1.5 (− 3.3, 0.29)	1.0 (− 3.8, 5.8)	0.33
36 months				
	**Mothers, Fathers, & Grandparents**^**a**^ **(*n*** **= 190, 100%)**	**Caregiver specific association**		
		**Mothers/Fathers** **(*n*** **= 138, 73%)**	**Grandparents** **(*n*** **= 52, 27%)**	**Interaction term** ***p*-value**
**MDI** **Log**_**2**_ **Hair THg (β)**	−0.23 (− 2.1, 1.6)	−1.6 (− 3.7, 0.54)	3.1 (− 0.21, 6.3)	0.02*
**PDI** **Log**_**2**_ **Hair THg (β)**	−1.0 (− 3.4, 1.3)	− 1.9 (− 4.7, 0.79)	1.2 (−3.0, 5.5)	0.21

*p < 0.05, **p < 0.01 *p*-values are for the Beta coefficients

*MDI* Mental Developmental Index, *PDI* Psychomotor Developmental Index, *THg* total mercury

Note: All estimates are from models that were adjusted for maternal age (years), maternal fish consumption (0 servings/weekly, 0 < servings/weekly< 2 servings/weekly, or ≥ 2 servings/weekly), maternal rice consumption (<daily, or ≥ daily), maternal serum zinc (μg/L), log_2_ maternal blood lead (μg/dL), log_2_ maternal energy intake (kcal), pre-pregnancy body mass index (underweight, normal weight, or overweight/obese), maternal education completed (<high school, high school, or some university), child sex, birth weight-for-gestational age (z-score), child’s primary caregiver (2 categories: mother/father, or grandparent), the difference in child’s age between the targeted age and actual age at testing (months), and the interaction between THg and the child’s caregiver

^a^This model does not include the interaction of THg with the child’s caregiver

Linear mixed models were re-run 1) including only the participants who returned at both times (*n* = 149); 2) using the raw Bayley scores; 3) including only participants with complete data (12 months: *n* = 234, 36 months: *n* = 169); 4) including only participants who did not eat fish (12 months: *n* = 109, 36 months: *n* = 82); 5) stratified by sex; 6) stratified by breastfeeding duration; and (7) without adjustment for covariates (See Table 4 and Supplementary Tables 4 and 5, Additional file 1). Overall findings in sensitivity analyses were not materially altered from primary analyses. In addition, at 12 months, there was suggestive evidence that boys, compared to girls, might be more sensitive to adverse MeHg associations with the MDI (β for boys = − 2.5, 95% CI: − 4.6, − 0.41; β for girls = − 1.2, 95% CI: − 3.2, 0.73) (See Supplementary Table 5, Additional file 1). At 12 months, adverse associations of MeHg with MDI were also stronger among children breastfed for less than the median duration (< 8.5 months) (β = − 2.1, 95% CI: − 4.2, − 0.08), compared to those breastfed longer (β = − 1.5, 95% CI: − 3.6, 0.61) (See Supplementary Table 5, Additional file 1). However, for MDI and PDI at both time periods, sex- or breastfeeding-specific estimates were imprecise and *p*-values for interactions between strata were 0.38–0.89.

## Discussion

Among rice consumers in rural China, prenatal MeHg exposure (estimated with log_2_ maternal hair THg) was associated with decrements in children’s cognitive function assessed with the BSID-II between 12 and 36 months of age. The overall association between MeHg and MDI scores was more adverse at 12 months compared to 36 months, while overall associations did not differ over time between MeHg and PDI scores. However, there was no evidence of attenuation of adverse MeHg associations over time in analyses subsetted on children whose primary care giver was a parent(s), which constituted most of the study cohort (91% at 12 months and 73% at 36 months) (Tables 1 and 6). Specifically, between 12 and 36 months, adverse associations were consistently observed (and modestly enhanced over time) among children raised by their parents (Table 6). Alterations in family structure between 12 and 36 months [with more parents leaving Daxin County for work and more children being cared for by grandparent(s)] modified associations between MeHg and BSID-II scores, and may have contributed to an apparent attenuation of MeHg associations over time in our data (Table 6).

In China, rapid development and urbanization has led to an increase in parental migration for work. In 2015, 29 million rural and urban children aged 0–5 years (30% of all children in that age range) were left-behind due to parental work migration [33]. A majority of left-behind children, especially younger children, are cared for by their grandparents [22, 33], who may be illiterate, lack parenting skills, or may be physically unable to care for a young child [34–36]. Conversely, the higher family incomes associated with having a migrant parent (or parents) may provide benefits to child development, including the potential for better nutrition among left-behind children under 5 years of age, compared to children living with both parents [37, 38]. In addition, higher household income for these families has been shown to contribute to improved housing conditions, and a more hygienic home environment, as well as greater utilization of health services [37], all of which could be beneficial to children’s neurodevelopment. In our longitudinal analysis, children cared for by a grandparent had lower MDI and PDI scores, compared to children cared for by their mothers (Table 4). This is consistent with findings from a large, longitudinal study (*n* = 1834 children) in rural Shaanxi Province, China, investigating the effects of maternal migration for work on children’s development [39]. Children were followed periodically between 6 and 30 months; those cared for mainly by their grandmothers had lower BSID-I MDI scores compared to children cared for by mothers who never migrated for work, while PDI scores did not differ [39].

In our cohort, in addition to having lower BSID-II scores, study children who were cared for by grandparents had altered susceptibility to MeHg (Table 6). For example, at 12 months, associations between MeHg and MDI were more adverse for children cared for by grandparents (β = − 5.7, 95% CI: − 9.8, − 1.6), compared to children cared for by parents (β = − 1.3, 95% CI: − 2.9, 0.20). As described above, households where grandparents provide primary care may be at a disadvantage regarding a number of factors, such as child supervision and intellectual support [34–36]. In addition, study children cared for by grandparents at 12 months were breastfed for a significantly shorter duration, compared to children cared for by their parents (See Supplementary Table 2, Additional file 1). At 12 months, associations between MeHg and MDI scores were more adverse for children breastfed < 8.5 months, compared to children breastfed ≥8.5 months (See Supplementary Table 5, Additional file 1). Breast milk contains more than 200 fatty acids, including DHA [40], which may modify adverse associations between MeHg and children’s cognitive development [41]. Prior to 2019, the nutritional standard in China for formula for infants ages 6–12 months did not include DHA [42], suggesting even greater nutritional disadvantages of bottle-feeding in China, compared to populations with DHA-supplemented formula.

In contrast to the 12-month findings, at 36 months, we observed an unexpected beneficial association of MeHg with MDI (β = 3.1, 95% CI: − 0.21, 6.3), among children cared for by grandparents (Table 6). The positive association could be due to chance but also supports the possibility of residual negative confounding, for example, by diet or other lifestyle factors that co-vary with biomarkers of MeHg but are beneficial to neurodevelopment in this small subset of our population. At 36 months (compared to 12 months), there was a much greater proportion of migrant working parents (22% versus 3%) and primary care by grandparents (27% versus 9%) (Table 1). The more profound change in family structure at this age (compared to 12 months) could reflect a population stratum with altered confounding patterns relative to the overall cohort.

In studies where seafood was the primary dietary source of MeHg and the BSID-II was used, prenatal MeHg exposure was often higher than in the Daxin cohort, although associations between MeHg and Bayley scores were less consistent. Among 1265 20-month old children in the Seychelles, maternal hair THg averaged 3.92 μg/g (nearly 10-fold higher than our cohort, Table 2); however, no associations between prenatal MeHg exposure and Bayley MDI or PDI were observed [43]. In models adjusted for polyunsaturated fatty acids, including DHA and AA, an increase of 1 μg/g in hair THg was associated with a 0.06-point decrease (standard error: 0.08) in MDI scores, and a 0.02-point increase (standard error: 0.09) in PDI scores [43]. In the coastal and urban areas of the Tohoku district in Japan, the median maternal hair THg was 2.0–2.6 μg/g (Tatsuka et al. 2018) (4.9–6.3 times higher than our cohort, Table 2). Among 1016 children from this district, adverse associations between biomarkers of prenatal Hg exposure and MDI and PDI scores at 18 months were observed in adjusted models but associations were imprecise and CIs included the null [44]. Just one study had lower prenatal MeHg exposure (in Poland) compared to our cohort (2.2 times lower, based on maternal blood THg) [14], in which adverse MeHg-Bayley associations attenuated between 12, 24, and 36 months [45]. In a population where rice is the primary source of MeHg, our results suggest that adverse associations between MeHg and MDI (particularly among children cared for by their parents) are more readily ascertainable, despite lower prenatal MeHg exposure, compared to most populations where seafood is the primary MeHg source. This may be, in part, because confounding and potential effect modification by beneficial nutrients in fish is less prominent.

Although our longitudinal study has many strengths, there are some limitations to note. First, eligible mothers planned to stay in Daxin County for 12 months. Had we screened participants for longer follow-up, it is possible the attrition rate at 36 months would have been lower. Second, at 12 months, parental migration was inferred from the number of months the child lived with both parents, and separate information for each parent was not obtained, which may have resulted in misclassification of parents as migrants or non-migrants. In addition, the length and timing of parental absence may be important [39, 46, 47]; however, this information was not collected at 12 months, and only half the participants provided this information at 36 months (range of time away: 3–20 months, *n* = 22–23 responses). Lastly, this is a mostly rural, poor population, and although we adjusted for potential confounders, such as maternal diet and education, there is still the potential for unmeasured confounding to have biased results.

## Conclusions

In conclusion, for young children living in rural China, where rice consumption is the primary source of MeHg exposure, a biomarker of prenatal MeHg exposure was associated with decrements in cognitive function assessed between 12 and 36 months of age. These associations were demonstrated at MeHg exposure levels well below most prior studies among seafood consumers, in which the Bayley Scales were assessed. However, changes in the family structure over the study follow up time interval potentially impacted children’s sensitivity to prenatal MeHg exposure.

## Supplementary Information

**Additional file 1.** Detailed Laboratory Methods, Detailed Methods for the Food Frequency Questionnaire, Supplementary Tables 1–5, Supplementary Figures 1–2, and References.

## Data Availability

All data generated or analyzed during this study are included in this published article and its supplementary information files.

## Abbreviations

AA: Arachidonic acid
BMI: Body mass index
BSID-II: Bayley Scales of Infant Development, 2nd Edition
DHA: Docosahexaenoic acid
EPA: Environmental Protection Agency
FFQ: Food frequency questionnaire
GAM: Generalized additive model
MDI: Mental Developmental Index
MeHg: Methylmercury
N-3 fatty acids: Docosahexaenoic acid, eicosapentaenoic acid, and alpha-linolenic acid
N-6 fatty acids: Linoleic acid and arachidonic acid
Pb: Lead
PDI: Psychomotor Developmental Index
RMB: Ren min bi
SD: Standard deviation
Se: Selenium
THg: Total mercury
Zn: Zinc
