# Supplementary material for: Maternal methylmercury exposure through rice ingestion and child neurodevelopment in the first three years: a prospective cohort study in rural China
Source: Environ Health. 2021 Apr 28;20:50. doi: 10.1186/s12940-021-00732-z (PMC8082930; doi:10.1186/s12940-021-00732-z)
Supplement: Supplementary file 1 — Additional file 1. Detailed Laboratory Methods, Detailed Methods for the Food Frequency Questionnaire, Supplementary Tables 1–5, Supplementary Figures 1–2, and References. [file 12940_2021_732_MOESM1_ESM.docx]

Additional File 1: MATERNAL METHYLMERCURY EXPOSURE THROUGH RICE INGESTION AND CHILD NEURODEVELOPMENT IN THE FIRST THREE YEARS: A PROSPECTIVE COHORT STUDY IN RURAL CHINA

Sarah E. Rothenberg, Susan A. Korrick, Jihong Liu, Yanfen Nong, Hua Nong, Chuan Hong, Eva P. Trinh, Xu Jiang, Fred J. Biasini, Fengxiu Ouyang

Table of Contents

Detailed Laboratory Methods pg. 2-3

Detailed Methods for the Food Frequency Questionnaire pg. 4

Supplementary Table 1 pg. 5

Supplementary Table 2 pg. 6-10

Supplementary Table 3 pg. 11-12

Supplementary Table 4 pg. 13-14

Supplementary Table 5 pg. 15

Supplementary Figure 1 pg. 16

Supplementary Figure 2 pg. 17-18

References pg. 19-20

Detailed Laboratory Methods

A. Methylmercury (MeHg)

Detailed methods are from Hong et al. (2016). Rice samples were ground into a powder using a coffee grinder, which was cleaned with ethanol after each sample to prevent carry-over of mercury. Rice methylmercury (MeHg) was extracted following Liang et al. (1996). Briefly, ~0.5 g rice was digested in 2 mL of 25% (w/v) potassium hydroxide-methanol for 3 h at 75 °C, then 6 mL of dichloromethane and 1.5 mL hydrochloric acid were added, samples were shaken for 30 min, centrifuged (4000 rpm = 3000 × *g*, 30 min), and the phases were separated (Whatman, 1PS). Milli-Q H_2_O (>18.0 MΩ cm^-1^) was added to 25 mL, and samples sat overnight. The following day, samples were heated for 1.5 h in a water bath at 60-70 °C to expel dichloromethane, and the volume was raised using Milli-Q H_2_O to 40 mL. Digests were analyzed using EPA Method 1630 [U.S. Environmental Protection Agency (U.S. EPA) 2001], including ethylation with sodium tetraethylborate, purge and trap onto Tenax traps, and quantification using gas chromatography-cold vapor atomic fluorescence spectrometry (Brooks Rand Model III, Seattle, WA, USA).

B. Total Mercury (THg)

Total mercury (THg) concentrations in maternal hair and fish tissue were analyzed following U.S. EPA Method 7473 (2007) using atomic absorption spectrometry (Lumex Model RA-915+/PYRO-915+, St. Petersburg, Russia).

C. Other Metals

Blood lead (Pb) levels were analyzed directly using graphite furnace- atomic absorption spectrometry (Cao et al. 2014) (PinAAcle 900Z, PerkinElmer, Waltham, Mass., USA). Serum zinc (Zn) and selenium (Se) concentrations were analyzed by inductively coupled plasma-mass spectrometry (Agilent 7500CE, USA), following U.S. EPA 3050B (U.S. EPA 1996)

D. Fatty acids

Maternal serum fatty acids (omega-3: docosahexaenoic acid and eicosapentaenoic acid; omega-6: alpha-linolenic acid, linoleic acid and arachidonic acid) were assessed by gas-liquid chromatography (Agilent 6890N-5975B with flame ionization detector). Peak retention times were identified by injecting known standards of >99% purity.

Detailed Methods for the Food Frequency Questionnaire

At study enrollment, mothers also completed a modified semi-quantitative 102-item Food Frequency Questionnaire (FFQ). Food categories included rice, seven categories of fish and shellfish (freshwater fish, ocean fish, shrimp, eel, snails, crab, and other shellfish), and other foods (e.g., pork, vegetables). For each food item, mothers selected from eight intake frequencies, ranging from “never or rarely” to “≥2 times/day,” and servings per day were summed for each food group. Aside from rice, the FFQ did not elicit serving size. For rice, mothers estimated quantity per serving by selecting one of three bowls from a picture or actual bowls. We assumed 170 g/serving for ocean fish and freshwater fish [U.S. FDA (U.S. Food and Drug Administration) 2001], and 100 g/serving for other seafood categories (Cheng et al. 2009). Serving sizes for other food groups were assigned based on Cheng et al. (2009). Energy intake (kcal) and the proportion of calories from fat, carbohydrates and protein were calculated from the Chinese Food Composition Tables (Yang 2009; Yang et al. 2005).

Supplementary Table 1. Quality assurance/quality control, including recovery of standard reference materials and matrix spikes, the relative standard deviation between replicate analyses, and detection limits (Hong et al. 2016; Rothenberg et al. 2016).

|  | **% Recovery** | | | | | | | **% RSD** |  |
| --- | --- | --- | --- | --- | --- | --- | --- | --- | --- |
| **Matrix** | **IAEA-086**  **(Human Hair)**  **Mean ± SD**  **(n)** | **NIST 1515**  **(Apple Leaves)**  **Mean ± SD (n)** | **NRC-TORT2 (Lobster)**  **Mean ± SD**  **(n)** | **Contox**  **Heavy Metals**  **(Blood)**  **Range**  **(n)** | **Seronorm**  **Trace Elements**  **(Blood)**  **Range**  **(n)** | **Contox**  **Trace Metals**  **(Serum)**  **Range**  **(n)** | **Matrix spikes**  **(250 pg)**  **Mean ± SD (n)** | **Mean ± SD**  **or Range**  **(n)** | **Detection limits** |
| **Hair THg** | 87 ± 6.7%  (52) | 89 ± 5.7%  (51) | NA | NA | NA | NA | NA | 5.5 ± 4.1% (369) | 0.0095  μg/g |
| **Fish tissue**  **THg** | NA | 91 ± 7.2%  (4) | NA | NA | NA | NA | NA | 4.2 ± 3.9% (13) | 0.001  μg/g |
| **Rice MeHg** | NA | NA | 96 ± 9.5%  (32) | NA | NA | NA | 96 ± 24%  (56) | 7.7 ± 5.1% (56) | 0.002  ng/g |
| **Blood Pb** | NA | NA | NA | 85-115%  (20) | 85-115%  (20) | NA | NA | <20%  (397) | 0.1  μg/dL |
| **Serum Zn** | NA | NA | NA | NA | NA | 85-115%  (20) | NA | <20%  (397) | 30  μg/L |
| **Serum Se** | NA | NA | NA | NA | NA | 85-115%  (20) | NA | <20%  (397) | 1.6  μg/L |

IAEA (International Atomic Energy Agency), MeHg (methylmercury), n (sample size), NA (not applicable), NIST (National Institute of Standards and Technology), NRC (National Research Council), Pb (lead), RSD (relative standard deviation = 100*SD/mean of replicate analyses), Se (selenium), THg (total mercury), Zn (zinc)

Supplementary Table 2. Comparison of maternal/child characteristics by child's primary caregiver, including mother, father, or grandparent.

|  | **12 months (n=264)** | | | | **36 months (n=190)** | | | |
| --- | --- | --- | --- | --- | --- | --- | --- | --- |
|  | **Mother**  **n (%)** | **Father**  **n (%)** | **Grandparent**  **n (%)** | **p-value** | **Mother**  **n (%)** | **Father**  **n (%)** | **Grandparent**  **n (%)** | **p-value** |
| **Mother's Age Upon Enrollment (years)** |  |  |  |  |  |  |  |  |
| Age < 20 | 17 (8) | 1 (3) | 2 (8) | 0.33 | 8 (7) | 2 (9) | 4 (8) | 0.93 |
| 20 ≤ Age < 30 | 117 (56) | 20 (63) | 18 (75) |  | 63 (55) | 14 (61) | 30 (58) |  |
| 30 ≤ Age < 45 | 74 (36) | 11 (34) | 4 (17) |  | 44 (38) | 7 (30) | 18 (35) |  |
| **Mother's Ethnicity** |  |  |  |  |  |  |  |  |
| Zhuang | 181 (87) | 28 (88) | 20 (83) | 0.37 | 102 (89) | 19 (83) | 44 (85) | 0.63 |
| Han | 23 (11) | 2 (6) | 4 (17) |  | 10 (9) | 4 (17) | 7 (13) |  |
| Other | 4 (2) | 2 (6) | 0 (0) |  | 3 (3) | 0 (0) | 1 (2) |  |
| **Mother's Education Completed** |  |  |  |  |  |  |  |  |
| < High School | 157 (75) | 27 (84) | 22 (92) | 0.29 | 87 (76) | 16 (70) | 43 (83) | 0.38 |
| High School | 34 (16) | 3 (9) | 1 (4) |  | 15 (13) | 5 (22) | 8 (15) |  |
| Some University | 14 (7) | 1 (3) | 0 (0) |  | 10 (9) | 1 (4) | 1 (2) |  |
| Missing | 3 (1) | 1 (3) | 1 (4) |  | 3 (3) | 1 (4) | 0 (0) |  |
| **Father's Education Completed** |  |  |  |  | 115 | 23 | 52 |  |
| < High School | 154 (74) | 26 (81) | 21 (88) | 0.72 | 83 (72) | 16 (70) | 41 (79) | 0.59 |
| High School | 35 (17) | 5 (16) | 3 (13) |  | 18 (16) | 5 (22) | 9 (17) |  |
| Some University | 15 (7) | 1 (3) | 0 (0) |  | 12 (10) | 1 (4) | 2 (4) |  |
| Missing | 4 (2) | 0 (0) | 0 (0) |  | 2 (2) | 1 (4) | 0 (0) |  |
| **Mother's Occupation** |  |  |  |  |  |  |  |  |
| Farmer | 149 (72) | 25 (78) | 16 (67) | 0.52 | 83 (72) | 15 (65) | 42 (81) | 0.31 |
| Worker**^a^** | 21 (10) | 2 (6) | 2 (8) |  | 15 (13) | 4 (17) | 5 (10) |  |
| Unemployed | 25 (12) | 0 (0) | 3 (13) |  | 13 (11) | 1 (4) | 3 (6) |  |
| Other | 9 (4) | 5 (16) | 3 (13) |  | 2 (2) | 2 (9) | 2 (4) |  |
| Missing | 4 (2) | 0 (0) | 0 (0) |  | 2 (2) | 1 (4) | 0 (0) |  |
| **Father's Occupation** |  |  |  |  |  |  |  |  |
| Farmer | 148 (71) | 24 (75) | 16 (67) | 0.68 | 77 (67) | 16 () | 41 (79) | 0.38 |
| Worker**^a^** | 33 (16) | 3 (9) | 4 (17) |  | 26 (23) | 2 (9) | 2 (4) |  |
| Unemployed | 12 (6) | 4 (13) | 2 (8) |  | 5 (4) | 2 (9) | 6 (12) |  |
| Other | 12 (6) | 1 (3) | 2 (8) |  | 5 (4) | 2 (9) | 2 (4) |  |
| Missing | 3 (1) | 0 (0) | 0 (0) |  | 2 (2) | 1 (4) | 1 (2) |  |
| **Household Monthly Income (RMB)^b^** |  |  |  |  |  |  |  |  |
| Income < 2000 | 126 (61) | 20 (63) | 16 (67) | 0.46 | 66 (57) | 17 (74) | 33 (63) | 0.53 |
| 2000 ≤ Income < 5000 | 54 (26) | 10 (31) | 4 (17) |  | 33 (29) | 3 (13) | 13 (25) |  |
| Income ≥ 5000 | 9 (4) | 0 (0) | 2 (8) |  | 5 (4) | 1 (4) | 3 (6) |  |
| Missing | 19 (9) | 2 (6) | 2 (8) |  | 11 (10) | 2 (9) | 3 (6) |  |
| **Maternal Pre-Pregnancy BMI (kg/m^2^)** |  |  |  |  |  |  |  |  |
| Underweight | 56 (27) | 10 (31) | 3 (13) | 0.046* | 34 (30) | 7 (30) | 10 (19) | 0.55 |
| Normal Weight | 110 (53) | 22 (69) | 17 (71) |  | 57 (50) | 14 (61) | 34 (65) |  |
| Overweight | 34 (16) | 0 (0) | 3 (13) |  | 21 (18) | 2 (9) | 7 (13) |  |
| Obese | 7 (3) | 0 (0) | 1 (4) |  | 3 (3) | 0 (0) | 1 (2) |  |
| Missing | 1 (<1) | 0 (0) | 0 (0) |  | 0 (0) | 0 (0) | 0 (0) |  |
| **Maternal Smoking During Pregnancy** |  |  |  |  |  |  |  |  |
| No | 204 (98) | 32 (100) | 24 (100) | NA | 113 (98) | 22 (98) | 51 (98) | 0.40 |
| Yes | 0 (0) | 0 (0) | 0 (0) |  | 0 (0) | 0 (0) | 1 (2) |  |
| Missing | 4 (2) | 0 (0) | 0 (0) |  | 2 (2) | 1 (4) | 0 (0) |  |
| **2nd-Hand Smoke Exposure During Pregnancy** |  |  |  |  | 115 | 23 | 52 |  |
| No | 120 (58) | 18 (56) | 11 (46) | 0.43 | 69 (60) | 13 (57) | 32 (62) | 0.98 |
| Yes | 81 (39) | 13 (41) | 13 (54) |  | 42 (37) | 9 (39) | 20 (38) |  |
| Missing | 7 (3) | 1 (3) | 0 (0) |  | 4 (3) | 1 (4) | 0 (0) |  |
| **Alcohol During Pregnancy** |  |  |  |  |  |  |  |  |
| No | 204 (98) | 30 (94) | 24 (100) | 0.06 | 112 (97) | 22 (96) | 49 (94) | 1.0 |
| Yes | 1 (<1) | 2 (6) | 0 (0) |  | 2 (2) | 0 (0) | 1 (2) |  |
| Missing | 3 (1) | 0 (0) | 0 (0) |  | 1 (<1) | 1 (4) | 2 (4) |  |
| **Anemia During Pregnancy** |  |  |  |  |  |  |  |  |
| No | 203 (98) | 29 (91) | 22 (92) | 0.05* | 112 (97) | 23 (100) | 49 (94) | 0.45 |
| Yes | 5 (2) | 3 (9) | 2 (8) |  | 3 (3) | 0 (0) | 3 (6) |  |
| **Primipara** |  |  |  |  |  |  |  |  |
| No | 96 (46) | 14 (44) | 9 (38) | 0.58 | 55 (48) | 10 (43) | 23 (44) | 0.86 |
| Yes | 101 (49) | 18 (56) | 15 (63) |  | 56 (49) | 11 (48) | 28 (54) |  |
| Missing | 11 (5) | 0 (0) | 0 (0) |  | 4 (3) | 2 (9) | 1 (2) |  |
| **Maternal Rice Consumption** |  |  |  |  |  |  |  |  |
| < Daily | 28 (13) | 4 (13) | 3 (12) | 1.0 | 18 (16) | 1 (4) | 5 (10) | 0.29 |
| ≥ Daily | 169 (81) | 25 (78) | 20 (83) |  | 90 (78) | 20 (87) | 46 (88) |  |
| Missing | 11 (5) | 3 (9) | 1 (4) |  | 7 (6) | 2 (9) | 1 (2) |  |
| **Maternal Fish Consumption (servings/week)** |  |  |  |  |  |  |  |  |
| 0 servings/week | 81 (39) | 15 (47) | 13 (54) | 0.40 | 48 (42) | 10 (43) | 24 (46) | 0.83 |
| 0 < servings/week < 2 | 102 (49) | 16 (50) | 9 (38) |  | 54 (47) | 11 (48) | 20 (38) |  |
| ≥ 2 servings/week | 25 (12) | 1 (3) | 2 (8) |  | 13 (11) | 2 (9) | 8 (15) |  |
| **Cesarean Birth** |  |  |  |  |  |  |  |  |
| No | 150 (72) | 26 (81) | 18 (75) | 0.61 | 86 (75) | 20 (87) | 41 (79) | 0.48 |
| Yes | 58 (28) | 6 (19) | 6 (25) |  | 29 (25) | 3 (13) | 11 (21) |  |
| **Child Gender** |  |  |  |  |  |  |  |  |
| Male | 97 (47) | 18 (56) | 9 (38) | 0.40 | 66 (57) | 14 (61) | 24 (46) | 0.35 |
| Female | 111 (53) | 14 (44) | 15 (63) |  | 49 (43) | 9 (39) | 28 (54) |  |
| **Gestational Age (weeks)** |  |  |  |  |  |  |  |  |
| 37 ≤ Gestational Age < 39 | 70 (34) | 15 (47) | 11 (46) | 0.29 | 45 (39) | 7 (30) | 18 (35) | 0.30 |
| 39 ≤ Gestational Age < 41 | 118 (57) | 17 (53) | 12 (50) |  | 61 (53) | 12 (52) | 32 (62) |  |
| Gestational Age ≥ 41 | 17 (8) | 0 (0) | 1 (4) |  | 7 (6) | 4 (17) | 2 (4) |  |
| Missing | 3 (1) | 0 (0) | 0 (0) |  | 2 (2) | 0 (0) | 0 (0) |  |
| **Birth weight-for-gestational age z-score (centile)** |  |  |  |  |  |  |  |  |
| Value < 10th | 23 (11) | 5 (16) | 4 (17) | 0.65 | 13 (11) | 3 (13) | 9 (17) | 0.72 |
| 10th ≤ Value < 90th | 172 (83) | 26 (81) | 18 (75) |  | 93 (81) | 20 (87) | 41 (79) |  |
| Value ≥ 90th | 10 (5) | 1 (3) | 2 (8) |  | 7 (6) | 0 (0) | 2 (4) |  |
| Missing | 3 (1) | 0 (0) | 0 (0) |  | 2 (2) | 0 (0) | 0 (0) |  |
| **Questionnaire Responses at 12 or 36 Months** |  |  |  |  |  |  |  |  |
| **At least one parent works outside Daxin County** |  |  |  |  |  |  |  |  |
| No | 204 (98) | 30 (94) | 7 (29) | <0.001*** | 104 (90) | 21 (91) | 24 (46) | <0.001*** |
| Yes | 1 (<1) | 1 (3) | 17 (71) |  | 11 (10) | 2 (9) | 28 (54) |  |
| Missing | 3 (1) | 1 (3) | 0 (0) |  | 0 (0) | 0 (0) | 0 (0) |  |
| **Older child in the household** |  |  |  |  |  |  |  |  |
| No | 125 (60) | 18 (56) | 17 (71) | 0.53 | 60 (52) | 13 (57) | 29 (56) | 0.93 |
| Yes | 83 (40) | 14 (44) | 7 (29) |  | 53 (46) | 10 (43) | 23 (44) |  |
| Missing | 0 (0) | 0 (0) | 0 (0) |  | 2 (2) | 0 (0) | 0 (0) |  |
| **Breastfeeding Duration > Median (8.5 months)^c^** |  |  |  |  |  |  |  |  |
| No | 94 (45) | 12 (38) | 21 (88) | 0.001*** | 55 (48) | 8 (35) | 26 (50) | 0.44 |
| Yes | 113 (54) | 20 (63) | 3 (13) |  | 59 (51) | 15 (65) | 26 (50) |  |
| Missing | 1 (<1) | 0 (0) | 0 (0) |  | 1 (<1) | 0 (0) | 0 (0) |  |
| **Child attends preschool** |  |  |  |  |  |  |  |  |
| No | NA | NA | NA | NA | 39 (34) | 12 (52) | 23 (44) | 0.17 |
| Yes | NA | NA | NA |  | 76 (66) | 11 (48) | 29 (56) |  |
| **Reported child illness in the previous 12 months^d^** |  |  |  |  |  |  |  |  |
| No | NA | NA | NA | NA | 12 (10) | 8 (35) | 9 (17) | 0.011* |
| ≥ 1 illness (range: 1-4) | NA | NA | NA |  | 103 (90) | 15 (65) | 43 (83) |  |
| **Child fish consumption within previous 24 hours** |  |  |  |  |  |  |  |  |
| No | NA | NA | NA | NA | 103 (90) | 18 (78) | 47 (90) | 0.30 |
| Yes | NA | NA | NA |  | 12 (10) | 5 (22) | 5 (10) |  |

*p ≤ 0.05, **p<0.01, *** p≤0.001, p-values are for chi-squared test or Fisher's exact test.

BMI (body mass intake), RMB (ren min bi = Chinese currency)

^a^Workers include: civil servant, white-collar worker, skilled worker, unskilled worker, and shopkeeper.

**^b^**Between 2013-2014, 2000 RMB = US$324, US5000 RMB = $810

^c^Median breastfeeding duration based on 12-month and 36-month responses (n=332 mothers).

^d^Illnesses included upper respiratory, lower respiratory, difficulty breathing, diarrhea, vomiting, fever, and rash. No differences were observed for specific illnesses (p=0.07-1.0).

Supplementary Table 3. Comparison of Bayley Scales of Infant Development-II, maternal biomarkers and diet, and child's anthropometry by child's primary caregiver, including mother, father, or grandparent (12 months: n=264 children, 36 months: n=190 children).

|  | **12 months (n=264)** | | | | **36 months (n=190)** | | | |
| --- | --- | --- | --- | --- | --- | --- | --- | --- |
|  | **Mother**  **Median**  **(range)** | **Father**  **Median**  **(range)** | **Grandparent**  **Median**  **(range)** | **p-value** | **Mother**  **Median**  **(range)** | **Father**  **Median**  **(range)** | **Grandparent**  **Median**  **(range)** | **p-value** |
| **n** | **208** | **32** | **24** |  | **115** | **23** | **52** |  |
| **Bayley standardized MDI** | 101  (66, 120) | 99  (77, 116) | 98  (77, 116) | 0.60 | 87  (70, 104) | 84  (62, 106) | 84  (63, 102) | 0.056 |
| **Bayley standardized**  **PDI** | 86  (60, 118) | 89  (61, 121) | 87  (74, 105) | 0.99 | 92  (71, 122) | 85  (75, 116) | 88  (75, 122) | 0.03* |
| **Maternal biomarkers and diet** |  |  |  |  |  |  |  |  |
| **Hair THg**  **(μg/g)** | 0.41  (0.12, 1.7) | 0.34  (0.18, 0.98) | 0.31  (0.08, 0.96) | 0.25 | 0.37  (0.12, 1.7) | 0.54  (0.19, 1.3) | 0.47  (0.15, 1.1) | 0.29 |
| **Rice MeHg**  **(ng/g)** | 2.1  (0.32, 15) | 2.0  (0.52, 12) | 2.5  (0.58, 9.2) | 0.39 | 2.1  (0.54, 11) | 1.3  (0.46, 4.6) | 2.2  (0.32, 15) | 0.09 |
| **n** | **206** | **32** | **24** |  | **114** | **23** | **52** |  |
| **%MeHg intake from rice** | 78  (0.30, 100) | 94  (9.0, 100) | 100  (9.7, 100) | 0.18 | 79  (0.32, 100) | 79  (20, 100) | 95  (0, 100) | 0.69 |
| **n** | **208** | **32** | **24** |  | **115** | **23** | **52** |  |
| **Serum Zn**  **(μg/L)** | 715  (344, 905) | 744  (599, 837) | 698  (572, 796) | 0.10 | 723  (575, 922) | 701  (638, 905) | 727  (586, 960) | 0.41 |
| **Blood Pb**  **(μg/dL)** | 2.6  (1.1, 7.8) | 2.6  (1.4, 4.9) | 2.6  (0.96, 5.1) | 0.58 | 2.7  (1.2, 7.6) | 2.5  (1.5, 7.8) | 2.8  (1.1, 7.3) | 0.84 |
| **Serum DHA**  **(mg/mL)** | 0.09  (0.04, 0.33) | 0.08  (0.05, 0.16) | 0.09  (0.06, 0.14) | 0.28 | 0.09  (0.05, 0.33) | 0.10  (0.06, 0.20) | 0.09  (0.04, 0.18) | 0.58 |
| **Serum EPA**  **(mg/mL)** | 0.007  (0.001, 0.09) | 0.006  (0.002, 0.08) | 0.007  (0.002, 0.02) | 0.43 | 0.008  (0.002, 0.07) | 0.007  (0.002, 0.03) | 0.007  (0.002, 0.03) | 0.42 |
| **Serum N-6/N-3**  **(unitless)** | 11  (3.5, 25) | 12  (5.3, 23) | 12  (6.9, 20) | 0.79 | 11  (5.0, 25) | 12  (5.7, 18) | 12  (5.9, 22) | 0.79 |
| **n** | **207** | **32** | **24** |  | **115** | **23** | **52** |  |
| **Serum Se**  **(μg/L)** | 153  (66, 535) | 157  (100, 248) | 152  (70, 251) | 0.73 | 153  (77, 323) | 152  (89, 320) | 153  (69, 264) | 0.98 |
| **n** | **199** | **32** | **24** |  | **108** | **22** | **52** |  |
| **% Calories from fat** | 33  (13, 81) | 33  (20, 74) | 33  (23, 69) | 0.73 | 33  (13, 77) | 29  (21, 59) | 33  (19, 81) | 0.15 |
| **% Calories from carbohydrates** | 55  (12, 75) | 55  (18, 72) | 56  (24, 68) | 0.97 | 56  (16, 78) | 60  (32, 69) | 56  (12, 68) | 0.10 |
| **% Calories from protein** | 12  (5.7, 25) | 11  (6.1, 17) | 10  (5.1, 17) | 0.05* | 12  (5.7, 17) | 11  (5.9, 15) | 11  (5.1, 22) | 0.63 |
| **Total Energy Intake (kcal)** | 2085  (549, 4438) | 1912  (606, 3631) | 1935  (602, 3945) | 0.08 | 1942  (557, 4637) | 2106  (809, 3169) | 2095  (549, 4069) | 0.61 |
| **Child anthropometry** |  |  |  |  |  |  |  |  |
| **n** | **207** | **32** | **24** |  | **115** | **23** | **52** |  |
| **Child Weight-for-age**  **(z-score)** | -0.58  (-2.71, 2.38) | -0.80  (-2.92, 0.24) | -0.61  (-1.58, 0.65) | 0.04* | -0.57  (-2.80, 2.51) | -1.11  (-2.21, 0.88) | -0.64  (-2.34, 1.31) | 0.13 |
| **Child Length/height-for-age (z-score)** | -0.58  (-3.25, 2.25) | -0.78  (-2.05, 1.11) | -0.85  (-2.84, 0.76) | 0.39 | -0.57  (-2.46, 1.79) | -0.56  (-2.27, 1.02) | -0.74  (-2.32, 1.28) | 0.64 |
| **Child Weight-for length/height (z-score)** | -0.38  (-2.46, 2.47) | -0.69  (-2.99, 0.33) | -0.24  (-1.48, 0.76) | 0.02* | -0.34  (-2.40, 3.54) | -0.81  (-1.78, 0.92) | -0.33  (-2.27, 1.41) | 0.08 |

*p ≤ 0.05, **p<0.01, *** p≤0.001, p-values are for Kruskal Wallis test.

DHA (docosahexaenoic acid), EPA (eicosapentaenoic acid), MeHg (methylmercury), N-6 fatty acids (linoleic acid and arachidonic acid), N-3 fatty acids (DHA, EPA and alpha-linolenic acid), Pb (lead), Se (selenium), THg (total mercury), Zn (zinc).

Supplementary Table 4. Sensitivity analyses assessing adjusted regression coefficients (95% confidence interval) associating log_2_ maternal hair total mercury with the Bayley Scales of Infant Development 2^nd^ Edition, from linear mixed models, including exam time specific associations .

For participants returning at both time points

|  |  | **Time Specific Associations** | | |
| --- | --- | --- | --- | --- |
|  | **All observations^a^**  **(n=298)** | **12 months**  **(n=149)** | **36 months**  **(n=149)** | **Interaction term**  **p-value** |
| **MDI**  **Log_2_ Hair THg (β)** | -1.7 (-3.4, -0.07)* | -2.0 (-4.1, 0.01)* | -1.4 (-3.4, 0.64) | 0.61 |
| **PDI**  **Log_2_ Hair THg (β)** | -1.8 (-3.6, -0.01)* | -2.1 (-4.4, 0.27) | -1.5 (-4.0, 1.0) | 0.72 |

For all participants, replacing the standardized Bayley scores with the raw Bayley scores

|  |  | **Time Specific Associations** | | |
| --- | --- | --- | --- | --- |
|  | **All observations^a^**  **(n=454)** | **12 months**  **(n=264)** | **36 months**  **(n=190)** | **Interaction term**  **p-value** |
| **MDI**  **Log_2_ Hair THg (β)** | -0.56 (-1.1, -0.05)* | -0.58 (-1.1, -0.03)* | -0.47 (-1.4, 0.44) | 0.82 |
| **PDI**  **Log_2_ Hair THg (β)** | -0.33 (-0.66, 0.004) | -0.29 (-0.67, 0.09) | -0.43 (-1.0, 0.17) | 0.69 |

For participants with complete data

|  |  | **Time Specific Associations** | | |
| --- | --- | --- | --- | --- |
|  | **All observations^a^**  **(n=403)** | **12 months**  **(n=234)** | **36 months**  **(n=169)** | **Interaction term**  **p-value** |
| **MDI**  **Log_2_ Hair THg (β)** | -1.3 (-2.5, -0.004)* | -1.6 (-3.1, -0.08)* | -0.75 (-2.6, 1.1) | 0.45 |
| **PDI**  **Log_2_ Hair THg (β)** | -1.0 (-2.5, 0.43) | -1.2 (-2.9, 0.62) | -0.76 (-3.1, 1.6) | 0.79 |

For participants who did not eat fish^b^

|  |  | **Time Specific Associations** | | |
| --- | --- | --- | --- | --- |
|  | **All observations^a^**  **(n=191)** | **12 months**  **(n=109)** | **36 months**  **(n=82)** | **Interaction term**  **p-value** |
| **MDI**  **Log_2_ Hair THg (β)** | -0.89 (-2.5, 0.69) | -1.8 (-3.7, 0.13) | 0.66 (-1.8, 3.1) | 0.11 |
| **PDI**  **Log_2_ Hair THg (β)** | -1.6 (-3.4, 0.18) | -1.9 (-4.1, 0.26) | -1.1 (-4.0, 1.8) | 0.64 |

*p≤0.05, p-values are for the Beta coefficients

MDI (Mental Developmental Index), PDI (Psychomotor Developmental Index), THg (total mercury)

Note: All estimates are from models that were adjusted for maternal age (years), maternal fish consumption (0 servings/weekly, 0<servings/weekly<2 servings/weekly, or ≥2 servings/weekly), maternal rice consumption (<daily, or ≥daily), maternal serum zinc (μg/L), log_2_ maternal blood lead (μg/dL), log_2_ maternal energy intake (kcal), pre-pregnancy body mass index (underweight, normal weight, or overweight/obese), maternal education completed (<high school, high school, or some university), child sex, birth weight-for-gestational age (z-score), child's caregiver (3 categories: mother, father, or grandparent), the difference in child's age between the targeted age and actual age at testing (months), a categorical variable for time (12 or 36 months), and the interaction between THg and exam time.

^a^This model does not include the interaction of THg with exam time.

^b^Estimates are from models that were adjusted for all covariates listed in the Note above, excluding maternal fish consumption

Supplementary Table 5. Adjusted regression coefficients (95% confidence interval) associating log_2_ maternal hair total mercury with the Bayley Scales of Infant Development, 2^nd^ Edition, including associations specific to child sex and breastfeeding duration.

12 months

|  |  | **Sex Specific Associations** | | |  | **Breastfeeding Specific Associations** | | |
| --- | --- | --- | --- | --- | --- | --- | --- | --- |
|  | **All**  **Children^a^ (n=264)** | **Male**  **(n=124)** | **Female**  **(n=140)** | **Interaction**  **p-value** | **All**  **Children^a,b^ (n=264)** | **Breastfed**  **<8.5 months**  **(n=127)** | **Breastfed**  **≥8.5 months**  **(n=137)** | **Interaction**  **p-value** |
| **MDI Log_2_ Hair THg (β)** | -1.8  (-3.3, -0.38)* | -2.5  (-4.6, -0.41)* | -1.2  (-3.2, 0.73) | 0.38 | -1.8  (-3.3, -0.37)* | -2.1  (-4.2, -0.08)* | -1.5  (-3.6, 0.61) | 0.66 |
| **PDI Log_2_ Hair THg (β)** | -1.2  (-2.9, 0.52) | -0.46  (-2.9, 2.0) | -1.8  (-4.1, 0.49) | 0.43 | -1.2  (-2.9, 0.51) | -0.56  (-3.0, 1.9) | -1.9  (-4.3, 0.60) | 0.46 |

36 months

|  |  | **Sex Specific Associations** | | |  | **Breastfeeding Specific Associations** | | |
| --- | --- | --- | --- | --- | --- | --- | --- | --- |
|  | **All Children^a^**  **(n=190)** | **Male**  **(n=104)** | **Female**  **(n=86)** | **Interaction**  **p-value** | **All Children^a,b^**  **(n=190)** | **Breastfed**  **<8.5 months**  **(n=90)** | **Breastfed**  **≥8.5 months**  **(n=100)** | **Interaction**  **p-value** |
| **MDI Log_2_ Hair THg (β)** | -0.17  (-2.0, 1.7) | 0.08  (-2.3, 2.5) | -0.51  (-3.3, 2.3) | 0.75 | -0.17  (-2.0, 1.7) | -0.04  (-2.7, 2.6) | -0.29  (-2.8, 2.2) | 0.89 |
| **PDI Log_2_ Hair THg (β)** | -0.89  (-3.2, 1.4) | -0.54  (-3.6, 2.5) | -1.4  (-4.9, 2.2) | 0.72 | -0.90  (-3.2, 1.4) | -1.4  (-4.7, 1.9) | -0.42  (-3.6, 2.8) | 0.67 |

*p<0.05, p-values are for the Beta coefficients

MDI (Mental Developmental Index), PDI (Psychomotor Developmental Index), THg (total mercury)

Note: All estimates are from models that were adjusted for maternal age (years), maternal fish consumption (0 servings/weekly, 0<servings/weekly<2 servings/weekly, or ≥2 servings/weekly), maternal rice consumption (<daily, or ≥daily), maternal serum zinc (μg/L), log_2_ maternal blood lead (μg/dL), log_2_ maternal energy intake (kcal), pre-pregnancy body mass index (underweight, normal weight, or overweight/obese), maternal education completed (<high school, high school, or some university), child sex, birth weight-for-gestational age (z-score), child's primary caregiver (3 categories: mother, father, or grandparent), the difference in child's age between the targeted age and actual age at testing (months), and the interaction between THg and child sex or between THg and child breastfeeding duration.

^a^This model does not include the interaction of THg with child sex or breastfeeding duration

^b^Estimates are from models that were adjusted for all covariates listed in the Note above, and for median breastfeeding duration (<median, ≥median, median=8.5 months)

Supplementary Figure 1. Sample size during enrollment, and at each follow-up visit (12 months and 36 months), including percentage returning (of 391 mother/child pairs eligible for this analysis).

Supplementary Figure 2. Partial residual plots from the covariate-adjusted semiparametric additive models for the Bayley Scales of Infant Development, 2^nd^ Edition and maternal hair total mercury (THg). Each plot describes the contribution of maternal hair THg to the developmental outcome, which has been centered to have a mean of zero (Axtell et al. 2000), including a) the Mental Developmental Index (MDI) and maternal hair THg at 12 months (n=264), b) the MDI and maternal hair THg at 36 months (n=190), c) the Psychomotor Developmental Index (PDI) and maternal hair THg at 12 months (n=263), and d) the PDI and maternal hair THg at 36 months (n=190). For figure c, one outlying observation was removed. The solid line = generalized additive model equation, and the dashed lines = the upper and lower pointwise twice-standard-error curves (degrees of freedom=4). The vertical marks along the x-axis indicate the observations. Models were adjusted for maternal age (years), maternal fish consumption (0 servings/weekly, 0<servings/weekly<2 servings/weekly, or ≥2 servings/weekly), maternal rice consumption (<daily, or ≥daily), maternal serum zinc (μg/L), log_10_ maternal blood lead (μg/dL), log_10_ maternal energy intake (kcal), reported pre-pregnancy body mass index (underweight, normal weight, or overweight/obese), maternal education completed (<high school, high school, or some university), child sex, birth weight-for-gestational age (z-score), child's caregiver (3 categories: mother, father, or grandparent), and the difference in child's age between the targeted age and actual age at testing (months).

REFERENCES

Axtell CD, Cox C, Myers GJ, Davidson PW, Choi AL, Cernichiari E, et al. Association between methylmercury exposure from fish consumption and child development at five and a half years of age in the Seychelles Child Development Study: an evaluation of nonlinear relationships. Environ Res Section A. 2000;84:71–80.

Cao J, Li M, Wang Y, Yu G, Yan CH. Environmental lead exposure among preschool children in Shanghai, China: blood lead levels and risk factors. PLoS One 2014; doi:10.1371/journal.pone.0113297

Cheng Y, Dibley MJ, Zhang X, Zeng L, Yan H. Assessment of dietary intake among pregnant women in a rural area of western China. BMC Public Health 2009; doi:10.1186/1471-2458-9-222.

Hong C, Yu X, Liu J, Cheng Y, Rothenberg SE. Low-level methylmercury exposure through rice ingestion in a cohort of pregnant mothers in rural China. Environ Res. 2016;150:519–27.

Liang L, Horvat M, Cernichiari E, Gelcin B, Balogh S. Simple solvent extraction technique for elimination of matrix interferences in the determination of methylmercury in environmental and biological samples by ethylation-gas chromatography-cold vapor atomic fluorescence spectrometry. Talanta 1996;43:1883–88.

Rothenberg SE, Yu X, Liu J, Biasini FJ, Hong C, Jiang X, et al. Maternal methylmercury exposure through rice ingestion and offspring neurodevelopment: a prospective cohort study. Int J Hygiene Environ Health. 2016;219:832–42.

U.S. Environmental Protection Agency (U.S. EPA). Method 3050B: Acid Digestion of Sediments, Sludges, and Soils, Revision 2. https://www.epa.gov/esam/epa-method-3050b-acid-digestion-sediments-sludges-and-soils (1996). Accessed 24 Feb 2021.

U.S. Environmental Protection Agency (U.S. EPA). Method 1630, Methyl Mercury in Water by Distillation, Aqueous Ethylation, Purge and Trap, and CVAFS. EPA-821-R-01-020. https://brooksapplied.com/wp-content/uploads/2013/12/1630.pdf. (2001). Accessed 24 Feb 2021.

U.S. Environmental Protection Agency (U.S. EPA). Method 7473 (SW-856), Mercury in Solids and Solutions by Thermal Decomposition, Amalgamation and Atomic Absorption Spectrophotometry. https://www.epa.gov/esam/epa-method-7473-sw-846-mercury-solids-and-solutions-thermal-decomposition-amalgamation-and (2007). Accessed 24 Feb 2021.

U.S. Food and Drug Administration (U.S. FDA). Advice About Eating Fish. https://www.fda.gov/food/consumers/advice-about-eating-fish (2001). Accessed 24 Feb 2021.

Yang Y, Wang G, Pan X. China Food Composition 2004 (Book 2), 1st ed. Beijing: Peking University Medical Center Press, ISBN: 978-7-81071-678-9; 2005.

Yang Y. China Food Composition (Book 1), 2nd ed. Beijing: Peking University Medical Center Press, ISBN: 978-7-81116-727-6; 2009.
